# Genome and Transcriptome-Wide Analysis of *OsWRKY* and *OsNAC* Gene Families in *Oryza sativa* and Their Response to White-Backed Planthopper Infestation

**DOI:** 10.3390/ijms232315396

**Published:** 2022-12-06

**Authors:** Ibrahim Khan, Rahmatullah Jan, Sajjad Asaf, Abdul Latif Khan, Saqib Bilal, Kyung-Min Kim, Ahmed Al-Harrasi

**Affiliations:** 1 Natural and Medical Sciences Research Center, University of Nizwa, Nizwa 616, Oman; 2 Division of Plant Biosciences, School of Applied Biosciences, College of Agriculture & Life Science, Kyungpook National University, Daegu 41566, Republic of Korea; 3 Department of Engineering Technology, University of Houston, Sugar Land, TX 77479, USA

**Keywords:** biotic stress, WBPH, transcription factors, *OsWRKY*, *OsNAC*, phylogeny, genomics

## Abstract

Plants are threatened by a wide variety of herbivorous insect assaults, and display a variety of inherent and induced defenses that shield them against herbivore attacks. Looking at the massive damage caused by the white-backed planthopper (WBPH), *Sogatella furcifera*, we undertook a study to identify and functionally annotate *OsWRKY* and *OsNAC* transcription factors (TFs) in rice, especially their involvement in WBPH stress. *OsWRKY* and *OsNAC* TFs are involved in various developmental processes and responses to biotic and abiotic stresses. However, no comprehensive reports are available on the specific phycological functions of most of the *OsWRKY* and *OsNAC* genes in rice during WBPH infestation. The current study aimed to comprehensively explore the *OsWRKY* and *OsNAC* genes by analyzing their phylogenetic relationships, subcellular localizations, exon–intron arrangements, conserved motif identities, chromosomal allocations, interaction networks and differential gene expressions during stress conditions. Comparative phylogenetic trees of 101 *OsWRKY* with 72 *AtWRKY* genes, and 121 *OsNAC* with 110 *AtNAC* genes were constructed to study relationships among these TFs across species. Phylogenetic relationships classified *OsWRKY* and *OsNAC* into eight and nine clades, respectively. Most TFs in the same clade had similar genomic features that represented similar functions, and had a high degree of co-expression. Some *OsWRKYs* (Os09g0417800 (*OsWRKY62*), Os11g0117600 (*OsWRKY50*), Os11g0117400 (*OsWRKY104*) and *OsNACs* (Os05g0442700, Os12g0630800, Os01g0862800 and Os12g0156100)) showed significantly higher expressions under WBPH infestation, based on transcriptome datasets. This study provides valuable information and clues about predicting the potential roles of *OsWRKYs* and *OsNACs* in rice, by combining their genome-wide characterization, expression profiling, protein–protein interactions and gene expressions under WBPH stress. These findings may require additional investigation to understand their metabolic and expression processes, and to develop rice cultivars that are resistant to WBPH.

## 1. Introduction

Plant stress is any unfavourable and non-optimal state that has detrimental effects on plant growth, development and crop yield by altering gene expression and cellular metabolism, which may lead to permanent damage or death if the stress exceeds the plant’s tolerance limits [1]. Plants are exposed to various environmental stresses, which can be classified as biotic stresses that are caused by plant pathogens, and abiotic stresses that are caused by environmental effects [2,3]. As one of the major sources of food and income, rice is cultivated worldwide. Rice flourishes in warm and humid climates, which are also conducive to pest proliferation. Pest infestation of rice crops adversely affects the growth rate and productivity of plants. Approximately 25% of global rice production is lost annually due to damage caused by pest assaults [4]. The white-backed planthopper (WBPH) is a serious insect pest that damages plants through cellsap-sucking and viral transmissions, and causes severe losses in yield each year, especially in Asian countries [5,6].

Similarly to other plant species, rice also developed sophisticated defense systems, such as releasing toxic compounds and generating plant resistance proteins that recognize pathogens and activate the plant defense responses through an effector-triggered immunity (ETI) process to cope with biotic stresses [7,8]. Due to rice’s scientific and economic importance, a concentrated study is required to understand the molecular mechanisms of innate immune responses in rice, and the intracellular signal transduction pathways involved in defense responses during biotic stress tolerance in rice.

Transcription factors (TFs) play essential roles in almost all cellular functions such as growth, development, metabolism, signal transduction and resistance to biotic and abiotic stress [9]. TFs are characterized proteins with at least one domain that dynamically regulates gene expression at the transcriptional level through recognizing specific cis-regulatory elements (CREs) in promoters, enhancers and other regulatory regions of the target genes in eukaryotic genomes [10,11,12]. Depending on the environment and target genes, TFs may act as activators or repressors of transcription, and may bind alone or in combination near the genes whose expression they regulate [13]. In order to regulate and coordinate the activation or repression of functional gene expression, TFs’ mediated responses are based on both internal and external signals [14]. TFs are identified and classified according to their structure and conserved motifs in DNA binding domains (DBDs) [15]. In plants, many transcription factors (TFs) act as key controllers of the multitudinous major growth and developmental processes, such as cellular morphogenesis and signal transduction during various environmental stresses [9,16]. Presently, 56 distinct TF families (about 7% of the coding part of plant transcriptomes) with 2408 members have been exclusively characterized in rice (*Oryza sativa*) and deposited in the PlantTFDB database [17]. Among these TFs, *WRKY* and *NAC* are the two most prominent expressed families, indicating their active involvement in plant growth, and serving as early stress-responsive factors against biological triggers at different developmental stages [10,18].

The *OsWRKY*, the seventh-largest family of TFs with 101 genes, is involved in the defense responses against biotic and abiotic stresses, and in developmental processes such as the production of secondary metabolites, hormone regulation, seed germination, pollen development and leaf senescence [19,20]. The *OsWRKY* proteins (*OsWRKYs*) are characterized by the *OsWRKY* domain that features a signature peptide sequence of approximately 60–70 amino acids residues with highly conserved amino acid motifs (*WRKY*GKK/*WRKY*GQK) for DNA binding promoter elements or W-Box (T)(T)TGAC(C/T) recognition at the N terminus, and for either a C_2_H_2_- or a C_2_HC-type zinc finger motif at the C-terminus of the sequence [21]. The *OsWRKYs* of higher plants, including rice, are categorized into three groups with several subgroups on the basis of phylogenies, and on the number and basic structure of *OsWRKY* domains (WDs) and types of zinc finger motifs [22,23].

The *OsNAC* family represents one of the largest families of plant-specific transcription factors, with 121 members in rice; the family has been proven to play important roles in regulating various plant processes and in stress responses [24,25]. All transcription factors of this family possess a conserved DNA-binding domain at the N-terminal (*OsNAC*), which comprises nearly 160 amino acid residues, and a very divergent transcriptional activation domain at the C-terminal involved in repressing or activating the transcription of multiple target genes [26]. *NAC* proteins have been demonstrated to participate in a wide range of plant developmental processes and plant responses to various biotic and abiotic stresses, such as embryo development, floral development, shoot apical meristem development, lateral root development, leaf senescence, cell cycle control, hormone signaling, grain nutrient remobilization and shoot branching determination [27]. Numerous *NAC* domain proteins have also been implicated in plant abiotic stresses and defense responses such as drought, salinity, cold shock, mechanical wounding and viral infection [28,29]. Many studies have shown that various *NAC* domain proteins are linked to plant defense and abiotic stresses, including drought, salinity, cold shock, abscisic acid (ABA) and mechanical injury [30].

The rice genome was sequenced and published for the first time in 2006, and has been considered a monocotyledonous model plant like maize, wheat sorghum and barley. It has been actively used by the plant research community in revolutionizing genetics and breeding studies [10,31]. Currently, there are 2408 genes that encode TFs in rice; some of them are essential for imparting biotic stress endurance to plants by activating the expression of stress-related genes, and synthesizing diverse functional proteins [2,32]. The *WRKY* and *NAC* TF families are reported to exhibit diverse functional roles in biotic stress tolerance. For instance, *OsWRKY45* conferred enhanced resistance against blast fungus [33]. *OsWRKY2*, *OsWRKY14*, *OsWRKY26*, *OsWRKY69* and *OsWRKY93* exhibited an alteration in transcriptional levels in response to *Magnaporthe grisea* infection [34]. *OsWRKY62* and *OsWRKY76* mediated defense against *Xanthomonas oryzae*, the causal agents of bacterial leaf blight [35,36]. *OsWRKY67* positively regulates blast fungus and bacteria blight resistance [37]. Overexpressions of *OsNAC6*, *OsNAC19* and *OsNAC111* regulate innate defense responses in rice against blast diseases, i.e., *Magnaporthe grisea* [38,39]. Expression of the *OsWRKY13* gene suppresses the JA-dependent pathway, while activating the SA-dependent pathway to mediate resistance against pathogenic bacteria and fungi [40]. However, no comprehensive reports have yet deciphered the specific physiological functions of most of these genes that regulate the immune response against biotic stress/pathogen infection in rice. Therefore, computational identification and functional annotation of TFs at the genomic scale are essential for understanding the mechanism of gene expression and regulation.

In this study, we comparatively characterized rice *OsWRKY* and *OsNAC* proteins based on incorporating phylogeny, chromosomal location, gene structure, conserved motifs and expression profiling. We also highlighted the potential role of these proteins in white-backed planthopper (WBPH) stress by analyzing their transcriptome profiling in response to WBPH stress. The current study provides a foundation for further comparative genomic studies. The inducibility pattern of diverse groups of genes with an increase in the duration of stress exposure is also undoubtedly valuable knowledge in stress genomics, especially for gene editing programs.

## 2. Results

### 2.1. Phylogenetic Analysis and Classification of OsWRKY and OsNAC Genes

**Figure 1:**
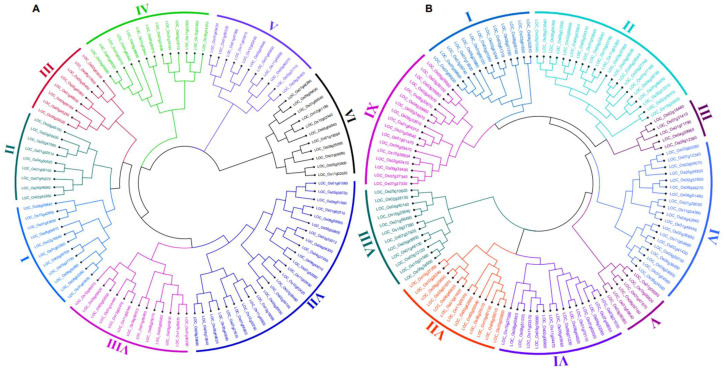
(**A**) The phylogenetic tree of 101 *Oryza sativa WRKY* TFs constructed via the NJ method using MEGA-11 software with 1000 bootstrap replicates. The major eight phylogenetic groups are marked as I to VIII, respectively. (**B**) The phylogenetic tree of 121 *Oryza sativa* NAC TFs constructed via the NJ method using MEGA-11 software with 1000 bootstrap replicates. The major nine phylogenetic groups are marked as I to IX, respectively.

To evaluate the phylogenetic and genealogical relationships among *OsWRKY* proteins, an unrooted phylogenetic tree was constructed from aligned deduced amino acid sequences by the NJ method with 1000 bootstrap replicates. As shown in Figure 1A, our phylogenetic analysis led to the classification of *OsWRKY* into eight major clades. Clade Ⅶ contained the maximum number, i.e., 25 (24.7%) of *OsWRKY* proteins, followed by clades Ⅳ and Ⅷ with 13 genes (12.87%) while, group III comprised the least number, i.e., 8 (7.9%) TFs. Similarly, based on the unrooted phylogenetic tree analysis, *OsNAC* TFs were divided into nine main clades (clades Ⅰ–Ⅸ) (Figure 1B). The data revealed that a maximum number, i.e., 21 (17.35%), of *OsNAC* members were found in clade Ⅱ, followed by clade Ⅳ with 19 (15.70%) members; notably, the minimum number, i.e., 5 (4%), was found in clade Ⅲ. Furthermore, most *OsWRKY* and *OsNAC* members within the same phylogenetic group had the same number of exons, and conserved exon–intron structure. For instance, 45% of the members of clades Ⅰ and Ⅱ contained three exons, and the majority of the members of clades Ⅳ and Ⅶ had rich intronic regions. These genes’ similar compositional and structural features may be related to their specific physiological functions in rice cells.

### 2.2. Phylogenetic Comparison of OsWRKY and OsNAC Proteins in Arabidopsis and Rice

**Figure 2:**
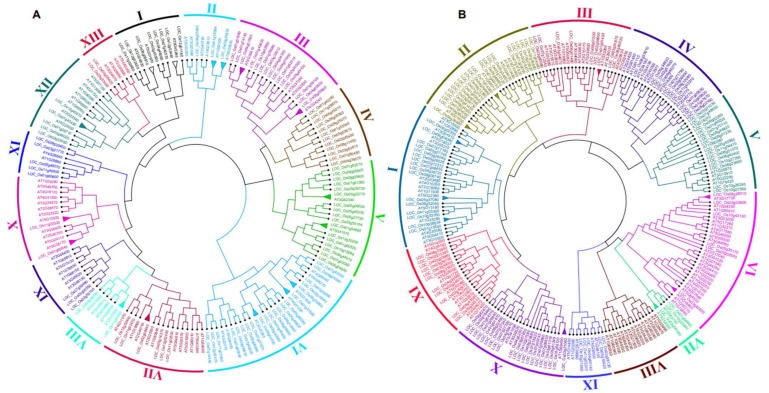
(**A**) Joint phylogenetic tree constructed from an alignment of 101 *Oryza sativa* (*OsWRKYs*) and 72 *Arabidopsis thaliana* (*AtWRKYs*) protein sequences via the NJ method with bootstrapping (1000 replicates) using MEGA-11 software. The resulting thirteen groups are shown in different colors. (**B**) Joint phylogenetic tree constructed from an alignment of 121 *Oryza sativa* (*OsNACs*) and 110 *Arabidopsis thaliana* (*AtNACs*) proteins sequences via the NJ method with bootstrapping (1000 replicates) using MEGA-11 software. The resulting eleven groups are shown in different colors.

To carefully infer the phylogenetic relationships of the rice *OsWRKY* and *OsNAC* TF families, *Arabidopsis,* the most extensively annotated species, was considered in the present study. A total of 101 *OsWRKY* and 121 *OsNAC* sequences were identified in rice, and 72 *AtWRKY* and 110 *AtNAC* were identified in *Arabidopsis* after the repetitive and redundant gene sequences were removed. In order to evaluate the evolutionary relationships, a joint phylogenetic tree for the *OsWRKY* transcription factor (TF) family was built from 173 collated rice and *Arabidopsis OsWRKY* genes using multiple alignment method in ClustalW (Figure 2A). Consistently with the unrooted tree produced by the neighbour-joining method, 13 groups (clades Ⅰ–XIII) were defined and further divided into sub-groups that were supported by high bootstrapping values. Both *OsWRKY* and *AtWRKY* genes were distributed in all clades except clade Ⅳ, which contained *OsWRKY* genes from rice only. The highest gene number (GN) was observed in clade Ⅵ (GN = 28), followed by clade Ⅲ, Ⅴ (GN = 19), clade Ⅶ (GN = 17), and clades Ⅹ and Ⅻ (GN = 14). Clade XIII was the smallest, with GN = 6.

An unrooted phylogenetic tree of the 121 *OsNAC* and 109 *AtNAC* TFs was constructed using multiple sequence alignment it was divided into 11 main clades (clades-Ⅰ–Ⅺ) (Figure 2B). The results showed that clade Ⅵ comprised the maximum number of genes (GN = 32), followed by clade Ⅰ (NG = 31), clade Ⅱ (NG = 27) and clade Ⅹ (NG = 24). Clade Ⅶ was the smallest, with NG = 5. Clade Ⅷ contained gene members from the *AtNAC* family only. In both TF family phylogenetic trees, the orthologous gene pairs identified by triangular shape were selected for subsequent analysis. A total of 16 orthologous gene pairs were obtained from the phylogenetic relationship of *OsWRKY* and *AtWRKY,* while only 10 orthologous gene pairs were detected in the combined tree of *OsNAC* and *AtNAC* (Figure 2A,B). It is worth stating that the majority of the phylogenetic groups and sub-groups that were defined were also supported by additional pieces of evidence, such as the rice and *Arabidopsis* individual trees of *OsWRKY* and *OsNAC* genes (Figure 1A,B), gene structure, protein–protein interaction, and physiological functions similarity of most of the characterized genes.

### 2.3. Orthology Relationships of OsWRKY and OsNAC Genes in Arabidopsis and Rice

**Figure 3:**
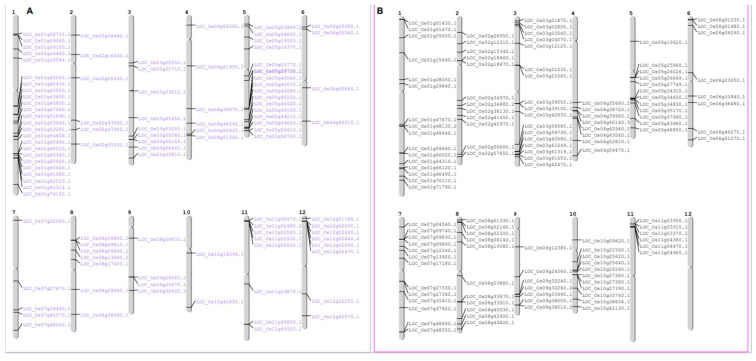
(**A**) Chromosomal localizations of *OsWRKY* genes on the twelve chromosomes of rice. (**B**) Chromosomal localizations of *OsNAC* genes on the twelve chromosomes of rice.

Orthologs are defined as genes in different genomes created by splitting taxonomic lineages that may retain the same function. The approach of orthology can identify the existence of genes involved in a common biological process, even if the expression data are insufficient to reveal the existence of co-regulatory networks [41]. To evaluate the evolutionary and orthology relationship within the *OsWRKY* and *AtWRKY* gene families, and within the *OsNAC* and *AtNAC* gene families, we performed combined phylogenetic analysis to obtain joint trees (Figure 2A,B). The tree topology and the group and sub-group organization resembled those from the rice and *Arabidopsis* individual trees of the *OsWRKY* and *OsNAC* genes (Figure 1A,B). We found 16 and 10 pairs of orthologous genes among rice and *Arabidopsis OsWRKY* and *OsNAC* TF families, respectively, as already displayed in their respective trees. In rice, the *OsWRKY* orthologous genes were distributed randomly in OsChr1, OsChr2, OsChr4, OsChr5, OsChr6, OsChr8, OsChr11 and OsChr12, while orthologs of *OsNAC* were found on OsChr1, OsChr3, OsChr5, OsChr6, OsChr9, OsChr10 and OsChr11 (Figure 3A,B).

### 2.4. Chromosomal Location and Gene Duplication

Out of 101 genes, 100 *OsWRKY* genes could be mapped on the chromosomes. The precise location of LOC_Os08g09900.1 could not be determined, while LOC_Os05g27730.1 and LOC_Os05g25770.1 were more likely to originate from the same ancestor gene during evolution of the rice chromosome. As represented in Figure 3A, 21 *OsWRKY* genes (22.34%) were located in chromosome 1, followed by chromosome 5 with 16 (17.02%) genes, chromosome 3 with 8 (8.51%) genes, and chromosomes 8 and 11 with 7 (7.44%) genes. Only a few *OsWRKY* genes were located on chromosomes 2, 6, 7, 9 and 10. As represented in Figure 3B, in rice the *OsNAC* genes were mapped from chromosomes 1 to 11, while no *OsNAC* gene was found on chromosome 12. There were 17 (14.04%) *OsNAC* genes mapped on chromosome 3, followed by chromosome 1 with 16 (13.22%), chromosome 7 with 13 (10.74%), and 12 (9.91%) genes were located on chromosomes 2 and 5.

**Figure 4:**
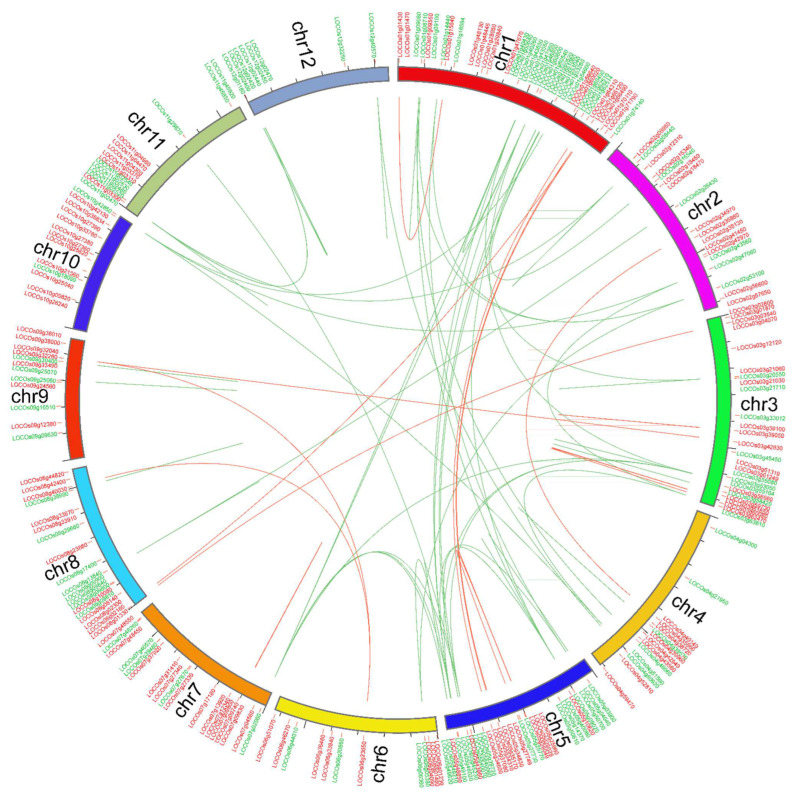
Chromosomal positions and inter-chromosomal groups of duplicated *OsWRKY* and *OsNAC* gene pairs were mapped on the twelve rice chromosomes (Chr1–Chr12). The green and red lines represent the segmental or tandem duplication network zones among *WRKY* and *NAC* genes, respectively.

Gene duplications (i.e., segmental and tandem) are thought to be the main factor supporting the expansion and evolution of gene families in plants [42]. Hence, gene duplication procedures were assessed between *OsWRKY* and *OsNAC* genes (Figure 4). The gene duplication study showed that about 35 *OsWRKY* gene pairs were unevenly mapped on different chromosomes. Mainly, chromosome 5 had a maximum number, i.e., 20 of *OsWRKY* gene pairs, followed by chromosome 11 with 16 *OsWRKY* gene pairs. Only one *OsWRKY* gene pair was discovered on chromosome 8, while the LOC_Os10g42850 (*OsWRKY*2) gene mapped on chromosome 10 had three pairs, i.e., on chromosomes 1, 2 and 4. The results revealed that segmental duplications contributed to the expression of *OsWRKY* genes. Similarly, 11 *OsNAC* gene pairs were detected irregularly on different chromosomes in the rice genome. Similarly for *OsWRKYs*, a maximum number of *OsNAC* gene pairs were mapped on chromosomes 5 and 11.

### 2.5. Insights into Exon–Intron Arrangements

**Figure 5:**
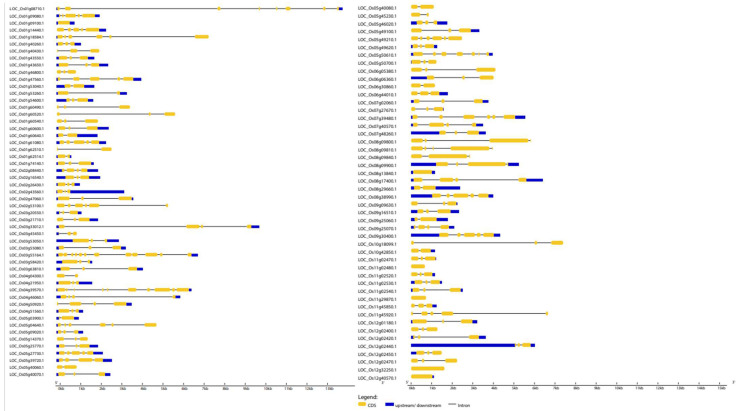
Exon–intron structure analysis of *Oryza sativa WRKY* genes was performed using the GSDS database. The blue boxes indicate upstream/downstream, the yellow boxes indicate exons, and the black lines indicate introns.

Gene structural similarity and diversity play a key role in gene family evolution [19]. Therefore, we generated separate exon–intron maps of the *OsWRKY* and *OsNAC* genes using the Gene Structure Display Server. Generally, the outcomes of the constructed phylogenetic trees for both *OsWRKYs* and *OsNACs* revealed that genes in the same clade shared similar intron/exon structures in points of exon number and length (Figure 5). For example, out of 11 *OsWRKY* genes in clade Ⅴ, 9 (82%) genes had three exons and 2 (18%) genes had five exons, and the majority of genes in clade Ⅶ contained more than three exons. Inversely, clades Ⅳ and Ⅷ showed a large difference, with exon numbers ranging from 1–8. These results indicate that clade Ⅴ and clade Ⅶ are more conserved, while clades Ⅳ and Ⅷ could be related to the evolution of the *OsWRKY* family.

**Figure 6:**
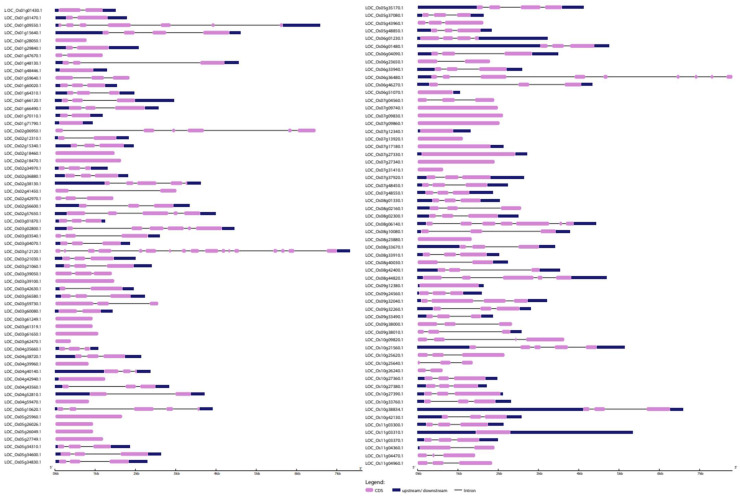
Exon–intron structure analysis of *Oryza sativa NAC* genes was performed with the GSDS database. The dark blue boxes indicate upstream/downstream, the purple boxes indicate exons, and the black lines indicate introns.

The detailed representation of the coding region, introns and upstream/downstream regions of the *OsWRKY* genes is provided in Figure 5. Introns, which are integral elements of eukaryotic genomes, actively participate in genomic recombination, leading to gene rearrangements and evolution. The outcomes revealed that most of the *OsWRKY* genes were rich in introns, and only six genes had no introns. The number of exons and introns varied from 1 to 12 and 0 to 11, respectively (Figure 5 and Table S1). The highest number of exons and introns, i.e., 12 and 11, respectively, were found in gene LOC_Os03g55164.1 (*OsWRKY4*), followed by LOC_Os04g39570.1 (*OsWRKY35*), with 10 exons and 9 introns, respectively, and LOC_Os1g08710-1(*OsWRKY*102) had 8 exons and 7 introns. Of 101 genes, 53(52%) *OsWRKY* genes had three exons and two introns. The genes LOC_Os1g08710-1 (*OsWRKY102*) and LOC_Os03g33012-1(*OsWRKY81*) had the largest intronic region, which shows their greater evolutionary conservation on the protein level. In contrast, most of the *OsNAC* genes had rich exonic regions; out of 121 genes, 30 *OsNAC* had no introns. The maximum numbers of exons and introns (17 and 16, respectively) were found in LOC_Os03g12120.1, followed by LOC_Os06g36480.1, with 9 exons and 8 introns, respectively (Figure 6). Out of 121 genes, 60 (about 50%) *OsNAC* genes had three exons and two introns. LOC_Os01g09550.1, LOC_Os02g06950.1, LOC_Os03g12120.1 and LOC_Os06g36480.1 had the largest intronic regions.

### 2.6. Analysis of Conserved Motifs

Apart from the conserved residues of the TFs, other motifs were also found in the rest of the protein sequence, which may perform unknown functional or structural roles. MEME analysis was performed to reveal the motifs’ structural and compositional similarity and diversity. The schematic distribution of these ten conserved motifs among *OsWRKY* and *OsNAC* proteins is shown in Figures S1 and S2. The blue-coloured motif (motif 1) was uniformly found in all of the *OsWRKY* proteins and significantly conserved the OsWRKY domain, while in the case of *OsNAC*, the red-colored motif (Motif1) was found in 90% of members, indicating that it was the *OsNAC* domain. We did not identify a potentially conserved motif (red) in 10% of *OsNAC* members. A possible reason may be that sometimes the training sequences are not strictly homologous, or contain repeated sequences, rearrangements, or other common situations that disrupt alignment approaches [43]. Our analysis showed that most evolutionary-related members in the constructed phylogenetic trees had common motif compositions; this indicates that the members of the *OsWRKY* and *OsNAC* families clustered in the same phylogenetic clade may be related to the same physiological functions. The structure of most of the genes from the same phylogenetic clade, as well as their motif compositions, were similar, e.g., members of clade Ⅲ and clade Ⅶ of the *OsWRKY* and *OsNAC* genes, respectively; this showed that the cladistics and classification of these two TF families in the present study were more reliable.

### 2.7. Protein Interaction Networks and Functional Annotations

**Figure 7:**
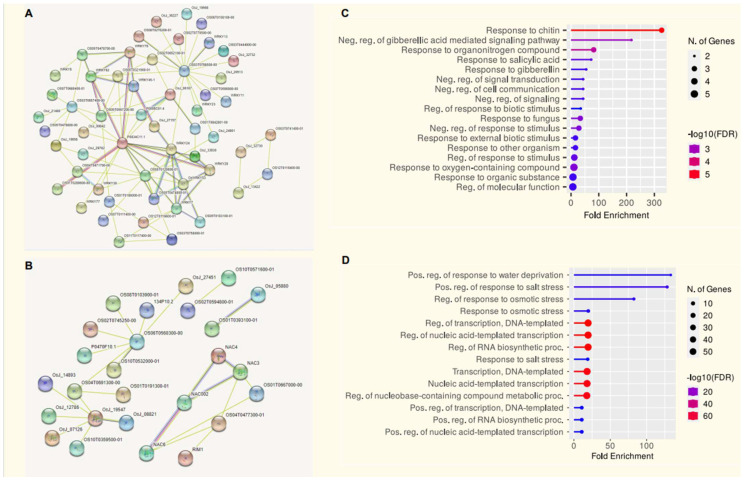
(**A**) Protein–protein association network of the *OsWRKY* genes based on their available information. The online tool STRING was used to predict the entire network. Different line colors represent the types of evidence for the associations, which are shown in the legend. (**B**) Protein–protein association network of the *OsNAC* genes based on their available information. The online tool STRING was used to predict the entire network. Different line colors represent the types of evidence for the associations, which are shown in the legend. (**C**) Gene ontology (GO) enrichment of the *OsWRKYs*. The sizes of circles indicate the number of genes in each category, while *x*-axis bars indicate fold enrichment. (**D**) Gene ontology (GO) enrichment of the *OsNACs*. The sizes of circles indicate the number of genes in each category, while *x*-axis bars indicate fold enrichment.

Multiple proteins can form homodimers or heterodimers that bind to DNA and regulate the transcription process of the targets; hence, protein–protein interactions have fundamental importance in gene expression. The putative *OsWRKY* and *OsNAC* protein sequences were added to the web-based system server STRING. The differentially expressed protein interaction network was built with default settings, except the organism, confidence (score), and no more than 10 interactors. The amino acid sequences of the TFs were input against the database, which contains all known and predicted protein–protein interactions. The retrieval included a detailed network of *OsWRKY* and *OsNAC* sequences, highlighting several hub proteins (Figure 7A,B and Table S2). Among the identified *OsNAC* proteins, only 10 had co-expression, while many *OsWRKY* proteins linked through direct interaction, and showed co-expression supported by high scores. Most of the interactions were also supported by the constructed phylogenetic tree. Thus, this profiling can aid in predicting the functions of the uncharacterized partner. For example, *OsWRKY45*, *OsWRKY62* and *OsWRKY76* express significantly for enhanced resistance against various biotic stresses [33,35,36]. However, no comprehensive reports are available on the specific physiological functions of *OsJ_06167* (*OsWRKY39*) and *OS05T0478700-00* (*OsWRKY84*); these two TFs shared protein–protein association networks with *OsWRKY45*, *OsWRKY62* and *OsWRKY76*, supported by a strong correlation score from the STRING analysis.

### 2.8. Gene Ontology Analysis of Rice OsWRKY and OsNAC Genes

To reveal the functional classifications of the *OsWRKY* and *OsNAC* genes, GO terms were predicted using the online tool Gene Ontology (GO). The results showed that the majority of *OsWRKY* genes were involved in responses to various stimuli, especially to biotic stresses such as organic substances (GO:1901576), oxygen-containing compounds (GO:1901700), organonitrogen compounds (GO:1901564) and chitin (GO:0010200) (Figure 7C); meanwhile, most of the *OsNAC* genes were involved in responses to abiotic stresses such as salinity (GO:0042538), water deprivation (GO:0009414) and osmotic stress (GO:0006970). Moreover, many *OsNACs* were found to be involved in cellular components such as DNA template transcription (GO:0006351) and RNA biosynthesis (GO:0032774) (Figure 7D).

### 2.9. Differential Gene Expression Analysis of OsWRKY and OsNAC Genes during WBPH Stress

**Figure 8:**
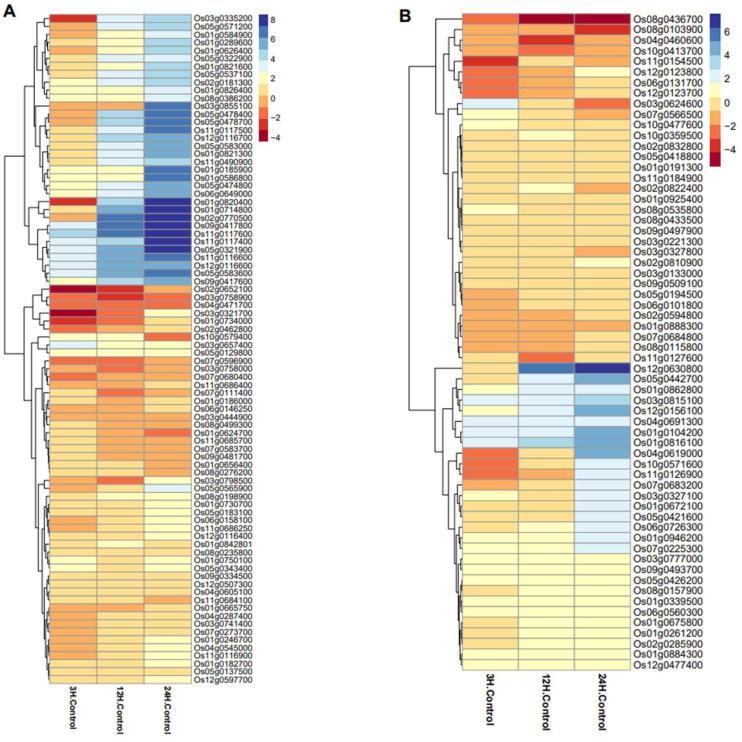
(**A**) Expression profiles of *OsWRKYs* in response to WBPH infestation at three different time points. Changes in gene expressions are shown in different colors. (**B**) Expression profiles of *OsNACs* in response to WBPH infestation at three different time points. Changes in gene expressions are shown in different colors.

We examined expression levels of *OsWRKY* and *OsNAC* genes during WBPH infestation. At different time points, both *OsWRKY* and *OsNAC* genes showed various responses. After inoculation with the WBPH pest, most of the genes showed high transcription levels. With a few exceptions, an increase in the duration of stress, resulted in maximum induced expression levels being recorded at 24 h of WBPH treatment. Some of the *OsWRKY* genes, such as Os09g0417800 (*OsWRKY62*), Os11g0117600 (*OsWRKY50*), Os11g0117400 (*OsWRKY104*), Os05g0321900 (*OsWRKY75*) and Os11g0116600 (*OsWRKY52*) were found to be highly upregulated, even after 3 h of infection; this regulation increased as the infection prolonged. Two *OsWRKY* genes, i.e., Os03g0335200 (*OsWRKY79*) and Os01g0820400 (*OsWRKY116*), were discovered to be significantly downregulated at the early stage of infection (3 h); however, as the infection proceeded, they showed upregulation. About 10 *OsWRKY* genes were found to be highly upregulated after 24 h of infection (Figure 8A). However, most of the *OsWRKY* genes showed downregulation during WBPH infestation. Contrary to the *OsWRKY* genes, only a few *OsNAC* genes, such as Os05g0442700, Os12g0630800, Os01g0862800, Os12g0156100, Os01g0104200, Os01g0816100 and Os04g0619000, showed upregulation during WBPH infection. However, eight *OsNAC* genes showed high downregulation after 3 h of infestation, and two genes, Os08g0436700 and Os08g0103900, were found to be highly downregulated (up to four-fold) after 24 h of infection (Figure 8B). Based on this late expression pattern, we suggest that these genes are involved in ETI signaling responses in rice-WBPH interaction. This information suggests that these genes may be involved in both ETI and pathogen-associated molecular pattern (PAMP)-triggered immunity (PTI) signaling response in rice-WBPH interaction. By contrast, the expression of one *OsWRKY* gene, Os03g0657400 (*OsWRKY*60), and one *OsNAC* gene (Os03g0624600), were downregulated at 12 and 2 h of WBPH infestation, respectively.

## 3. Discussion

As one of the major sources of food and income, rice is grown in more than 100 countries. The dramatic increase in human population constitutes a need to increase rice production by 70%, in order to fulfill upcoming demand by 2050 [8,44]. Rice grows in warm and humid climates, which are also conducive to pest proliferation. Pest infection has severe consequences on plant growth and productivity. The WBPH is an important migratory insect that feeds on phloem sap and spreads plant viruses; it causes significant damage to rice plants such as tillering delay, reduction in vigor, yellowing of leaves, shrivelling of grains and even causing death [45]. Besides virus transmission, WBPH harbours fungal (yeast) and bacterial (*Wolbachia*, *Cardinium*, *Asaia*) symbionts [46]. These symbionts are important for the host insect in termss of providing essential amino acids to their food, facilitating host digestion and enhancing insecticide resistance [47]. However, to the best of our knowledge, there is a lack of information which shows that WBPH transmits pathogenic bacteria to rice plants. For the current experiment, the WBPH were provided by the Rural Development Administration (RDA), South Korea, and were reared in a controlled environment. However, some rice varieties are resistant to WBPH [48]. Increasing research in this area aims to understand the mechanisms of this resistance. Rice plants have highly flexible adaptivity to adverse conditions such as exposure to biotic and abiotic factors that trigger various responses governed by complex regulatory mechanisms; through transcriptional regulation and gene expression, plants respond to these changes by either activating or repressing the expression of downstream genes [49,50]. Transcription factors (TFs) are deployed as the master key regulators in plant growth, development and defense-related responses. The *WRKY* and *NAC* are major TF families that regulate various aspects of plant development, growth and responses against biotic and abiotic stresses [38,51]. During the past few years, significant advancements have been made in studying the defensive mechanisms of *WRKY* and *NAC* genes against stress conditions; however, thorough examinations are required to study the specific physiological roles of most of these genes. *Abidopsis* is the most extensively studied plant used for most facets of plant molecular and evolutionary biology. In the current study, a genome-wide comparative analysis of *OsWRKY* and *OsNAC* genes was carried out with two major goals: to provide a foundation for the functional characterization of uncharacterized *OsWRKYs* and *OsNACs* TFs, and to open a new window for further functional studies on the roles of *OsWRKYs* and *OsNACs* under biotic stress, especially in WBPH infestation.

Combined phylogenetic trees were constructed to study the phylogenetic evolutionary relationships and orthologs of *OsWRKY* and *OsNAC* genes in *Arabidopsis* and rice genomes. In the *OsWRKY* phylogenetic tree, both the *Arabidopsis* and rice genes were present in virtually all clades (Figure 1). In contrast, the phylogenetic tree of *OsNAC* genes showed that clade Ⅷ was dominated by *Arabidopsis OsNAC* genes (Figure 2). Orthologous genes are similar genes with the same genetic function that may have arisen from speciation events. A relatively higher number of orthologous gene pairs were observed in the *Arabidopsis*-rice *OsWRKY* TF family, which may show that ancestral relationships existed between *Arabidopsis* and rice before their divergence during evolution. The arrangements of exons and introns also supported the phylogenetic analysis. Many of the *OsWRKY* and *OsNAC* TFs in the same clade displayed the same numbers of exons and introns, while some TFs belonging to the same clade displayed various exon and intron numbers. These results suggest that the genes with the same exon and intron numbers are more conserved and imply similar function, while genes of the same clade with different exon and intron numbers suggest that they may have undergone loss of introns during evolutionary processes. Conserved motif analysis indicated that all of the *OsWRKYs* harboured the typical *OsWRKY* domain, while the potentially conserved motif was not found in a few *OsNAC* members; these findings indicate situations such as non-homology, repetition or rearrangements in their sequences. Our outcomes confirmed that *OsWRKY* and *OsNAC* genes of rice suffered segmental duplications, indicating that the duplicated genes may play an essential role in gene expression and evolution [52]. To boost our understanding regarding the involvement of *OsWRKY* and *OsNAC* genes contrary to biotic stress, we examined expression levels of these genes during WBPH stress. Our results showed that after WBPH infestation, the majority of the *OsWRKYs,* such as Os09g0417800 (*OsWRKY62*), Os11g0117600 (*OsWRKY50*), Os11g0117400 (*OsWRKY104*) Os02g0770500 (*OsWRKY32*), Os01g0820400 (*OsWRKY116*), Os05g0321900 (*OsOsWRKY75*), Os03g0855100 (*OsWRKY80*), Os05g0478400 (*OsWRKY48*), Os05g0478700 (*OsWRKY84*)*, Os01g0821600 (OsWRKY24), Os08g0386200 (OsWRKY69), Os06g0649000 (OsWRKY28), Os02g0181300 (OsWRKY71), Os11g0490900 (OsWRKY72)* and some *OsNACs* such as (Os05g0442700, Os12g0630800, Os01g0862800 and Os12g0156100) were significantly upregulated in rice (Figure 8A,B), indicating that *OsWRKY* and *OsNAC* genes exhibit extensive responses to WBPH infestation. Our results are in agreement with those of previous studies showing that *OsWRKY62* plays a role in basal defense against *Xanthomonas oryzae pv. Oryzae* [33]. *OsWRKY50* mediates ABA-dependent seed germination [53], and the expression of *OsWRKY80* is strongly induced upon infection with *Rhizoctonia solani* [54]. *OsWRKY24* and *OsWRKY69* were reported to confer resistance against fungal pathogens, whereas *OsWRKY28* and *OsWRKY71* were reported for resistance against both bacterial and fungal pathogens [40]. It was previously reported that *OsWRKY72* plays an essential role in abiotic stress tolerance [55]; however, in the current study we found that *OsWRKY72* was expressed during WBPH infestation, suggesting its potential role in biotic stress. Similarly, an overexpression of *OsWRKY11* has been reported to enhance heat and drought tolerance [56]; however, the current study showed that it may also be involved in the response to WBPH infestation.

Essential clues and deep insights regarding the physiological functions of rice *OsWRKY* and *OsNAC* genes were obtained from their different expression patterns and interaction networks. The specific physiological functions of uncharacterized proteins are typically assigned through their alignments with homologous sequences of already-characterized genes of the same or other species. Therefore, a joint phylogenetic tree of rice with the more extensively annotated Arabidopsis was constructed in the present study. The already characterized *OsWRKY45* (LOC_Os05g25770) and uncharacterized *OsWRKY84* (LOC_Os05g40070) genes exhibited the same exon–intron arrangements, sub-cellular locations, motif compositions and protein interaction networks, and were located on the same chromosome; thus, we can predict the function of *OsWRKY84* and its potential involvement in inducing resistance against abscisic acid (ABA) stress and infection by [57] *oryzae* and *Xanthomonas oryzae* [57]. Similarly, the LOC_Os07g12340, LOC_Os03g60080 and LOC_Os06g36480 genes of the *OsNAC* family were found to belong to the same clade of the phylogenetic tree, located in the same sub-cellular location and present in same protein–protein interaction network. However, all three of these *OsNAC* genes were not characterized for their specific physiological roles, but our analysis showed that LOC_Os07g12340 (Os07g0225300) has high expression during WBPH infestation, so we can predict that it may be involved in biotic stress tolerance.

## 4. Materials and Methods

### 4.1. Plant Material, WBPH Infestation and RNA Extraction

We used the wild-type (WT) Nagdong rice cultivar in the current study. Rice seeds were sterilized and soaked for three days in an incubator at 32 °C in dark conditions. After successful sprouting, the seeds were separated and transferred to pots in a greenhouse. The greenhouse temperature was maintained at 30/28 °C during a 16/8 h light/dark photoperiod, and at a humidity of 60%. After two weeks, the plants were exposed to WBPH in the insectarum under the same growth conditions. About 10 plants per pot of each genotype were exposed to the insect, with a ratio of 10 WBPH per plant. The WBPH were starved for 2 h prior to the infestation in a beaker with wet tissue paper. Leaf samples were collected randomly after 0 h, 3 h, 12 h and 24 h of WBPH infestation. The wild type at 0 h was considered a control. RNA was isolated using an RNeasy plant mini kit (QIAGEN, Hilden, Germany); the quality was evaluated using Nano-drop 1000 (Thermo Scientific. Waltham, MA, USA), and the quantity was adjusted to 10 µg of total RNA.

### 4.2. Data Resources

Genes of *O. sativa* and *Arabidopsis WRKY* and *OsNAC* encoding transcription factors (TFs) were retrieved from the Plant Transcription Factor Database v5.0 (PlantTFDB 5.0; http://planttfdb.gao-lab.org/, accessed on 15 November 2022) [58]. The corresponding protein-coding sequences were obtained from Phytozome 12.1 (https://phytozome.jgi.doe.gov/pz/portal.html, accessed on 15 November 2022) [59].

### 4.3. OsWRKY and OsNAC Family Identification in O Sativa

The locus identifier IDs of *OsWRKY* and *OsNAC* proteins were subjected to Rice Genome Annotation Project (http://rice.uga.edu/index.shtml, accessed on 15 November 2022) to find their gene names, locus names, alternative splices from, chromosome numbers, nucleotide lengths (bp), predicted protein lengths (aa), predicted molecular weights (Da) and theoretical isoelectric points (pI). The subcellular localizations of *OsWRKY* and *OsNAC* proteins were predicted using CELLO (http://cello.life.nctu.edu.tw/, accessed on 15 November 2022) (Table S1).

### 4.4. Multiple Sequence Alignment and Phylogenetic Analysis

Multiple sequence alignment (MSA) was conducted using MEGA software [60]. The sequences were aligned with Clustal W, and the alignment parameters were included in pairwise alignments (gap opening penalty was 15.00, gap extension penalty was 6.66) and multiple alignments (gap opening penalty was 15.00, gap extension penalty was 6.66). Then, Neighbor-Joining (NJ) method was carried out with 1000 bootstrap replicates, while for other parameters, default settings of the softwarewere used to draw the phylogenetic tree. The phylogenetic tree was visualized and annotated using FigTree software v1.4.4 (http://tree.bio.ed.ac.uk/software/figtree/, accessed on 15 November 2022) [61]. 

### 4.5. Chromosomal Location and Gene Duplication Analysis

Chromosomal location analysis of the rice *OsWRKY* and *OsNAC* gene families was performed using the Oryza Base Chromosome Map Tool http://viewer.shigen.info/oryzavw/maptool/MapTool.do, accessed on 15 November 2022). The TFs with ≥70% similar aligned sequences of the entire gene length were defined as duplicated genes. Genes that were separated by less than five gene loci at 100 kb distance were considered tandem duplicates [62], and those which were found as co-paralogs and located on duplicated chromosome blocks were considered segmental duplicates [63]. To determine the physical locations of rice *OsWRKY* and *OsNAC* genes, the starting and ending positions of all the genes on each chromosome were obtained from the tomato database. MapInspect software was used to draw the images of the locations of the rice *OsWRKY* and *OsNAC* genes (http://mapinspect.software.informer.com/,accessed on 15 November 2022). We used the plant genome duplication database (PGDD, available at http://chibba.agtec.uga.edu/duplication/,accessed on 15 November 2022) to retrieve the duplicate chromosomal blocks, and then identify the *OsWRKY* and *OsNAC* genes in the duplication block; this allowed us to identify duplicate rice *OsWRKY* and *OsNAC* genes [64]. The PGDD is a public database used to identify and catalogue plant genes in terms of intra-genomic or cross-genomic syntenic relationships.

### 4.6. Exon–Intron Organization, Identification and Analysis of Conserved Motifs

A number of exons and introns and their structural features in *OsWRKY* and *OsNAC* TF genes were visualized using Gene Structure Display Server 2.0 (http://gsds.gao-lab.org/, accessed on 15 November 2022). The tool required the gene sequences and corresponding coding sequences as input. The conserved motifs of the target sequences were identified using Multiple Expectation Maximization for Motif Elicitation (MEME) Suite Software (http://meme-suite.org/, accessed on 15 November 2022). The maximum number of repetitions was set to any, the maximum number of motifs was set to 10, and the optimum motif width was set to >6 and <200 amino acids residues, in order to confirm the conserved domains of the candidate *OsWRKY* and *OsNAC* TFs.

### 4.7. Prediction of Protein–Protein Interaction Network

The putative *OsWRKY* and *OsNAC* protein sequences were added to the online server STRING version 11.0 (https://string-db.org/, accessed on 15 November 2022) to predict the protein–protein interaction networks.

### 4.8. Gene Ontology-Based Functional Annotation Analysis

Enrichment of gene ontology (GO) categories was performed with an agriGO analysis toolkit (http://systemsbiology.cau.edu.cn/agriGOv2/, accessed on 15 November 2022) [65], using the TopGO ’elim’ algorithm [66] for the ’biological process’ and ‘subcellular localization’ aspects.

### 4.9. Expression Analysis of Rice OsWRKY and OsNAC Genes in Response to WBPH Stress

RNA was isolated from fresh leaves after 0 h, 3 h, 12 h and 24 h of WBPH stress, with purification and library construction. The total RNA of each sample was diluted to 100 ng. The Illumina HiSeq2000 platform was used to execute the library construction procedure according to the approach described in [67], resulting in 51-base-pair single-end reads. Libraries from three different biological replicates of each treatment were sequenced and analyzed. A computational pipeline of optimized tools was employed to identify variations in gene regulation between the “inoculated” and “non-inoculated” plants. The pipeline included the following steps: (1) using FastQC (https://www.bioinformatics.babraham.ac.uk/projects/fastqc/, accessed on 15 November 2022) for the quality check; (2) using Trim Galore (Trim Galore) for data trimming; (3) using HISAT2 [68] for indexing and alignment to reference genome; (4) read count quantification using Feature Count (subread_v2.0.2); and (5) using DESeq2 [69] in the R program for differential gene expression analysis.

## 5. Conclusions

In conclusion, rice’s *OsWRKY* and *OsNAC* gene families were comprehensively analyzed to reveal gene structure, chromosomal allocations, gene duplications, phylogenetic relationships, conserved amino acid residues, and expression profiles during WBPH infestation. The expression analysis carried out on both *OsWRKY* and *OsNAC* genes implied that many of them are actively involved in inducing resistance against WBPH stress in rice plants. The current study provides valuable insights for the functional characterization of *OsWRKY* and *OsNAC* genes, and establishes a foundation for future studies on the production of disease-free rice varieties.

## Data Availability

The data presented in this study are available on request from the corresponding author.

## Supplementary Materials

The following supporting information can be downloaded at: https://www.mdpi.com/article/10.3390/ijms232315396/s1, Figure S1: The most conserved common motifs of *OsWRKY* TFs family were identified by MEME database with the complete amino acids sequences. The blue colored motif signifies the *WRKY* motif; Figure S2: The most conserved common motifs of *OsNAC* TFs family were identified by MEME database with the complete amino acids sequences. The red colored motif signifies the *NAC* motif; Table S1:The data of 101 *WRKY* and 121 *bHLH* genes identified in *Oryza sativa* genome; Table S2: The Accession IDs and STRING Identifier of the *WRKY* and *NAC* TFs in *Oryza sativa*.
