# Supplementary material for: Genome and Transcriptome-Wide Analysis of OsWRKY and OsNAC Gene Families in Oryza sativa and Their Response to White-Backed Planthopper Infestation
Source: Int J Mol Sci. 2022 Dec 6;23(23):15396. doi: 10.3390/ijms232315396 (PMC9739594; doi:10.3390/ijms232315396)
Supplement: Supplementary file 1 [file ijms-23-15396-s001.zip › Supplementary Table S2.pdf]

Co-expressed Proteins

	S.No	STRING Identifier	MSU IDs
OsWRKYs	1	OS01T0289600-00	LOC_Os01g18584.1
	2	P0034C11.1	LOC_Os01g14440.1
	3	OS01T0826400-01	LOC_Os01g61080.1
	4	OsWRKY53	LOC_Os05g27730.1
	5	OS04T0471700-00	LOC_Os01g14440.1
	6	OsJ_33930	LOC_Os11g29870.1
	7	WRKY28	LOC_Os06g44010.1
	8	WRKY7	LOC_Os05g46020.1
	9	OS05T0183100-01	LOC_Os05g09020.1
	10	OS05T0474800-01	LOC_Os05g39720.1
	11	OsJ_27157	LOC_Os08g29660.1
	12	OsJ_06167	LOC_Os02g16540.1
	13	WRKY45-1	LOC_Os05g25770.1
	14	WRKY76	LOC_Os09g25060.1
	15	WRKY62	LOC_Os09g25070.1
	16	OS05T0478700-00	LOC_Os05g40070.1
	17	WRKY24	LOC_Os01g62510.1
OsNACs	1	NAC4	LOC_Os01g60020.1
	2	NAC3	LOC_Os07g12340.1
	3	NAC002	LOC_Os03g60080.1
	4	NAC6	LOC_Os01g66120.1
	5	OsJ_05880	LOC_Os02g12310.1
	6	OS01T0393100-01	LOC_Os01g29840.1
	7	OS06T0560300-00	LOC_Os06g36480.1
	8	OS10T0532000-01	LOC_Os10g38834.1
	9	OsJ_19547	LOC_Os05g48850.1
	10	OsJ_08821	LOC_Os02g56600.1
